# Adaptive Evolution of a Stress Response Protein

**DOI:** 10.1371/journal.pone.0001003

**Published:** 2007-10-10

**Authors:** Tom J. Little, Lenny Nelson, Ted Hupp

**Affiliations:** 1 Institute of Evolutionary Biology, School of Biology, University of Edinburgh, Edinburgh, United Kingdom; 2 CRUK p53 Signal Transduction Group, University of Edinburgh, Edinburgh, United Kingdom; University of Cape Town, South Africa

## Abstract

**Background:**

Some cancers are mediated by an interplay between tissue damage, pathogens and localised innate immune responses, but the mechanisms that underlie these linkages are only beginning to be unravelled.

**Methods and Principal Findings:**

Here we identify a strong signature of adaptive evolution on the DNA sequence of the mammalian stress response gene SEP53, a member of the epidermal differentiation complex fused-gene family known for its role in suppressing cancers. The SEP53 gene appears to have been subject to adaptive evolution of a type that is commonly (though not exclusively) associated with coevolutionary arms races. A similar pattern of molecular evolution was not evident in the p53 cancer-suppressing gene.

**Conclusions:**

Our data thus raises the possibility that SEP53 is a component of the mucosal/epithelial innate immune response engaged in an ongoing interaction with a pathogen. Although the pathogenic stress mediating adaptive evolution of SEP53 is not known, there are a number of well-known candidates, in particular viruses with established links to carcinoma.

## Introduction

Stress responses classically involve heat shock proteins or molecular chaperones that maintain protein function or repair damage after cell injury. As such, the integrity of chaperone systems can critically alter the progression of diseases associated with ageing, DNA damage and chronic injury [1], [2]. Although the molecular chaperone proteins are among the most evolutionarily conserved proteins and have a ubiquitous function in all repair processes, there is a high degree of tissue specificity in chaperone induction [3], [4], [5], indicating that some cells have evolved unique stress responses due to unique microenvironmental pressures.

Surface squamous epithelium is one such example, as it is not buffered from environmental stresses by the circulatory system, and is subject to a range of relatively unique stresses including thermal stresses and, in the gut, refluxed acid and bile adducts. Squamous epithelia may additionally be subject to bacterial infestation and viruses, for example infection by Papilloma viruses which can lead to cervical cancer [6], [7], [8]. Recently a “functional proteomics” study showed that stressed squamous cells do not synthesize the classic stressed-induced protein HSP70 [9], but instead express a novel class of stress proteins [10], including the Squamous Epithelial induced stress Protein of 53 kDa (SEP53; synonyms include c1orf10 and cornulin). SEP53 was independently cloned as a gene expressed in normal oesophagus but down-regulated in oesophageal squamous cancers and was named Clone 1 open reading frame 10 [10]. The SEP53 gene is located on chromosome 1q21 within the epidermal differentiation complex fused-gene family that is silenced as part of a general mechanism that suppresses genes from this locus in cancer cells [11], [12]. Molecular characterization of SEP53 has demonstrated that its stress-responsive functions are linked to its activity as a survival factor. For example, death induced by exposure of cells to normally lethal levels of deoxycholic acid (DCA; a bile stress imposed upon the gastrointestinal tract) can be attenuated by SEP53 [13].

SEP53 may have additional functions, and because some epithelial cancers are linked to microbes, our particular interest was the role that SEP53 might play in the defense against pathogenic infection of epithelial tissue. A hallmark of genes involved in host-pathogen interactions is an exceptionally fast rate of protein evolution (corrected for mutation rate) [14], [15], [16], [17], [18]. This arises because fighting pathogens leads to arms races: a series of selective sweeps caused by repeated adaptation and counteradaptation between host and pathogen. Indeed, almost half the documented cases of arms races are pathogen defense genes [18], raising the possibility that the identification of molecular arms races provides a first approach to implicating a gene's involvement in defense. In the present study, comparisons between human and primate SEP53, as well as phylogenetic analyses on a broader range of mammal species, identified the signature of an arms race, suggesting that SEP53 could be engaged in antagonistic interactions with a pathogen. As we were unsure if a high rate of molecular evolution might be a general feature of cancer-associated genes, we performed a similar analysis on p53, but this gene, well known for its central role in a range of cancers in all tissue types, showed no evidence of rapid adaptive evolution.

## Results and Discussion

Analysis of SEP53 coding sequence across 9 mammalian species revealed that this gene evolves rapidly through positive selection. We compared the rate of replacement nucleotide substitutions (substitutions which result in an amino acid change, *K_A_*) to the rate of synonymous nucleotide substitutions (substitutions that do not result in an amino acid substitutions, *K_S_*, which evolve at an approximately neutral rate). As most genes are subject to purifying selection, i.e. where replacement mutations produce inferior phenotypes that are pruned from the population, *K_A_*/*K_S_* tends to be much less than 1.0. Reflecting this, mean *K_A_*/*K_S_* between species is typically much less than 0.2 [19], [20]. *K_A_*/*K_S_* at SEP53 is in the upper range of that seen in the vast majority of genes, especially in the C-terminal open reading frame (Table 1). For comparison, the p53 gene, also known to suppress cancers, but in a functionally dissimilar way, showed very low *K_A_*:*K_S_* ratios (Table 1).

**Table 1:** The stress response protein SEP53 evolves rapidly through positive natural selection as evidenced by an elevated K_A_∶K_S_ ratio.

	K_A_/K_S_			
Sep-53	All Species	Human-Chimpanzee	Human Orangutan	Human-Gorilla
Total	0.41	0.79	1.07	1.58
N-Terminal Domain	0.12	0.62	0.31	0.63
C-Terminal Domain	0.46	0.88	1.15	2.06
p53	0.18	0 (K_A_ = 0)	-	-

Table shows average pairwise K_A_/K_S_ for the two main functional regions of SEP53 across nine mammalian species (left-most numeric column) and for three particular pairwise comparisons. The p53 gene did not show an elevated K_A_:K_S_ ratio in any pairwise comparisons of fourteen species studied.

**Figure 1:**
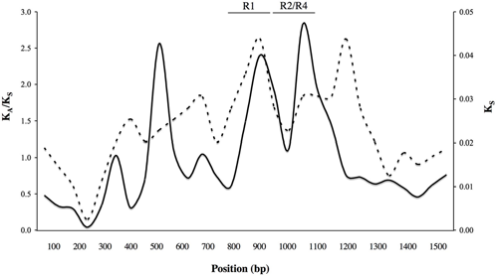
K_A_/K_S_ (solid line, left y-axis) varies greatly along the length of the SEP53 coding region based on a sliding window analysis. K_S_ (dotted line, right y-axis) is also shown. Data are from primates only and is the mean of all pairwise comparisons involving humans. The window size was 100 bp, the step size 20 bp; varying these parameters has some effect on the output, but all combinations of window and step size consistently indicated a K_A_/K_S_ trough in the N-terminal Ca+ binding domain (bp 0 to 270) and prominent peaks in the C-terminal domain (bp 271 onwards) around bp 500, 900, and 1100. The location of two repeats common to all primates are indicated as R1 and R2/R4 (see figure 2).

Whilst *K_A_*:*K_S_* ratios >>1.0 are evidence of positive selection (see also [21]), a simple *K_A_/K_S_* calculation is thought to be conservative because it represents the average ratio across all codons, and does not account for the possibility that some codons are highly conserved while others evolve rapidly. Indeed, sliding window analysis along the length of SEP53 showed depressed *K_A_/K_S_* in the highly conserved N-terminal Ca^+^ binding domain and a series of peaks in the C-terminal domain (Figure 1; full alignment of SEP53 is presented in supplementary information). We therefore used an approach that allows *K_A_/K_S_* to vary among codons to compare the likelihood of a model that permits a proportion of codons to be under selection to the likelihood of a model that assumes neutrality at all sites [22], [23], [24]. These comparisons of neutral and selection models indicated that SEP53 was subject to adaptive evolution (Table 2), with as many as 8 percent of codons showing *K_A_/K_S_* substantially above 1.0. For the same analysis on the p53 gene, *K_A_/K_S_* was low and neutral models were as likely as selection models (Tables 1, 2). Analysis of variation within the human population did not provide additional evidence of selection at SEP53 (or p53) (Table S1), however there was little power to do so as polymorphism at both loci was extremely low.

**Table 2:** Statistical tests for adaptive evolution indicate positive selection at the SEP53 locus.

	SEP53		P53
Models	2Δl	ω>1 (proportion)	2Δl
M1a vs M2a	11.6***	2.71 (0.08)	0.00
M8a vs M8	11.4***	3.48 (0.05)	0.20

Analysis used the computer program CODEML of the PAML package to compare models assuming K_A_/K_S_<1.0 at all sites (M1a, M7, M8a) to models assuming K_A_/K_S_>1.0 at some sites. 2Δl is two times the difference in the log likelihood for the two models under comparison. *** indicates p<0.0001. The column ‘ω>1’ shows K_A_/K_S_ in the category of sites estimated to be above 1.0 in the selection models (and in brackets the proportion of sites in that category).

**Figure 2:**
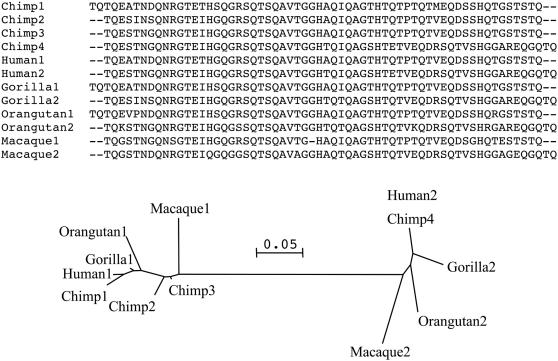
The SEP53 gene contains repeat regions, the chimpanzee has four of these and the other primates only two. A neighbour joining tree indicated that copy 4 in the chimpanzee was orthologous with copy 2 in the other species. Chimpanzee copy 1 is probably orthologous with copy 1 in the other species, but this was less clear.

Although the same domains appear to be present in all mammalian SEP53 open reading frames, the chimpanzee SEP53 gene contains a large insertion. Such a difference in amino acid sequence is unusual in *Pan*-*Homo* comparisons. SEP53 contains repeat regions, and the insertion sequence in *Pan* is two additional tandem repeats that other species lack (Figure 2). Many positively selected sites were in the repeat regions that the primates share, suggesting that these are evolutionary hotspots. Examination of the four tandem repeats in the chimpanzee offers further support for the action of positive selection. Assuming these repeats arise through duplication and that differences between repeats then accumulate independently (i.e. without concerted evolution), *K_A_/K_S_* between chimpanzee repeats averaged 1.07 and ranged up to 1.97. Additionally, we created a data set that included repeats from all primate SEP53's (*Pan* (four repeats), *Homo, Gorilla, Orangutan* and *Macaca* (two repeats each)). Average *K_A_/K_S_* in this data set was again high (0.89) and by using a gene tree of these repeats we performed maximum likelihood analyses as above to show that selection models were in all cases significantly more likely than neutral ones (for example, model 8 vs 8a, χ^2^ = 19.8, p<0.0001). Indeed, almost half of the sites showed evidence of positive selection. Thus, both SEP53 divergence between species and the diversification of repeats within SEP53 appears to be driven by positive selection.

The low *K_A_/K_S_* and lack of positively selected sites in the calcium binding domain (Table 1; Figure 1) indicates evolutionary constraint and that SEP53 functional activity relies strongly on the conservation of this domain. By contrast, the high K_A_:K_S_ ratio in the SEP53 C-terminal domain indicates that new mutations in some codons may be adaptive and selected up to high frequency. Past examples of adaptive evolution of this sort has been dominated by two classes of genes: i) genes associated with reproduction, including seminal fluid factors and proteins of the female reproductive tract [25]. The selective pressure on reproductive genes is likely to be driven by arms races linked to sexual conflict; ii) immune system genes engaged in an arms race of adaptation and counter-adaptation with parasites or pathogens. Such an anti-pathogen role is the most likely explanation for rapid evolution of SEP53. The epidermis is a notable point of pathogen entry, and indeed a regional immune response is well developed [26], [27], [28], [29], [30]. Thus, in addition to its role in mediating epidermal damage associated with adenocarcinoma, the pattern of adaptive evolution of SEP53 indicates that it is part of the epithelial immune response. We thus forward SEP53 as a candidate link between pathogens, innate immunity and epithelial cancers [6], [7], [8].

This raises intriguing possibilities regarding the role of SEP53 in attenuating stress and in cancer progression. Although the SEP53 protein's capacity to limit stress and control carcinogen-mediated DNA damage may be an important contribution to cancer avoidance, a pronounced role for SEP53 during mammalian evolution might have been to eliminate lethal squamous-cell viruses that effect both overall fitness and cancer progression. Playing a dual role as stress response protein and a viral defence molecule could limit SEP53's effectiveness at one of these tasks. For example, the need to coevolve with a pathogen could alter the protein to a degree that compromises stress-response functionality or the capacity to interact with other epithelial proteins, thus leading to cancer.

To conclude, much genomic research is centred on identifying homologous regions of a protein to acquire information on the essential function of a gene product. Recent whole-genome comparisons [20], [31], [32], [33] have, however, noted the rapidly evolving set of genes that play a role in speciation or are subject to rapid adaptive evolution. We speculate that the evidence for adaptive evolution in SEP53 reveals its role in immunity because the majority of cases of selective sweeps involve host-pathogen arms races. Thus, the present study highlights how documenting regions of high amino acid divergence can reveal hitherto un-thought-of roles for particular genes.

## Materials and Methods

Human SEP53 was used to search the non-redundant, Expressed Sequence Tag, and high throughput genomic sequences in EMBL/Genbank or the EMBL trace repository with Blast [34]. This revealed homologous sequences for a range of mammals, but none for non-mammalian species. Species obtained from Genbank and used in analyses were: human (*Homo sapiens*), pig (*Sus scrofa*), rat (*Rattus norvegicus*), mouse (*Mus musculus*), chimpanzee (*Pan troglodytes*), cow (*Bos taurus*), and the macaque (*Macaca fascicularis*).

We further isolated orangutan and gorilla SEP53 from genomic sequences obtained from the ECACC (European Collection of Cell Cultures). For this. we used the polymerase chain reaction (PCR) and oligonucleotide primers designed from human intronic sequence so that complete coding sequences were obtained for the SEP53 gene's two coding exons. One set of primers (FW: GAGCCTCCAAGGGAACTTTT RV; CTGCTATGTCCCCTCTCCAC) amplified the exon that codes for the EF-hand domain of SEP53, the other set (FW: GGATGCTGACTCCACCTCAT; RV:GCAGGACAAGCCAAACTCTC) amplified the exon corresponding to the C-terminal domain. PCR amplicons were electrophoresed, excised from agarose gels, cleaned and then sequenced (with the primers above plus additional internal ones to ensure that we had two fold coverage in all areas) in both directions using BigDye reagents and an ABI capillary sequencer. The sequence chromatograms were inspected by eye to confirm the validity of all differences within and between species, and assembled using SeqManII (DNAstar Inc., Madison USA).

Sequences were aligned with ClustalW and *K_A_* and *K_S_* were calculated using the program DNAsp [35]. We also used DNAsp to perform sliding window analysis along the length of the SEP53 sequence to visualise areas with high or low *K_A_*/*K_S_*. To statistically test for positive selection, we used the phylogeny-based analysis of *K_A_*/*K_S_* as implemented in the Codeml program of the PAML package [22], [23], [24]. Specifically, we varied the Nssites option to generate log likelihood values for models where *K_A_*/*K_S_* is specified to vary among sites, but can be constrained to be <1.0 (neutral models) or allowed to rise above 1.0 (selection models). We studied a range of neutral (denoted M1a, M7, and M8A) and selection models (M2a, M3 and M8) but focused attention on the comparisons thought to be the most robust (M1a vs M2a, M8a vs M8; [25], [36]). Significance was assessed using two times the difference in log-likelihood (2Δ*l*) value for each model, which is expected to follow a chi-squared distribution with degrees of freedom determined by the difference in the number of parameters for each model.

One notable feature of SEP53 was repeat regions of approximately 60 amino acids long (Figure 2). All of the primates except the chimp have two tandem repeats, while the chimp has four, leading to an insertion sequence of approximately 120 amino acids. We performed alignment and phylogenetic analysis separately on these repeat regions to ensure their accurate placement in the global SEP53 alignment (Figure 2, S1). Porcine SEP53 also contains an insert in this region (further details and functional analyses of porcine SEP53 are being presented elsewhere).

K_S_ ranged from 0.0095 (human-chimp) to 0.79 (Macaque-mouse). The latter is value is somewhat high, raising the possibility that some sites are saturated and thus our estimates of K_S_ may be inaccurate. We therefore repeated the analysis using only the five primates (mean K_S_ = 0.04). While this reduced data set lacked power due to the small number of species and slight divergence between them, the primate-only data set has the most confident alignment. The primate results confirmed the analysis of all nine species (for example, model 8 vs 8a, χ^2^ = 9.37, p<0.001). K_S_ also appeared to vary along the length of the SEP53 gene (Figure 1) and this is known to influence the detection of selection[37]. We tested for variation in K_S_ using the codon selection analysis component of the computer program HYPHY [38]. Whilst this did not indicate significant variation in K_S_ (two times the difference in log-likelihood between dual variable rates model and variable nonsynonymous rates model = 0.94, df = 4 p = 0.92), we nevertheless repeated the selection analysis (with all nine species) using the HyPHy/Datamonkey interface. Using the random effects likelihood method (REL) which allows for rate heterogeneity in both K_S_ and K_S_ and the TrN93 model (as chosen by the built in model selection tool), Datamonkey confirmed the results from PAML by identifying 20 positively selected sites in Sep53 (Bayes factor = 50).

The same analyses and comparisons between species were applied to the p53 gene. Fourteen sequences for phylogenetic analysis of the p53 gene were obtained from Genbank: *Homo sapiens, Pan troglodytes, Macaca fascicularis, Rattus norvegicus, Mus musculus, Bos taurus, Macaca fascicularis, Macaca mulatta, Macaca fuscata, Cavia porcellus, Spalax judaei, Sus scrofa, Ovis aries,* and *Mesocricetus auratus.*

Additional evidence for selection can be gained by comparing patterns of polymorphism within species to divergence between species. To obtain polymorphism data, we sequenced both SEP53 and p53 in 24 individuals from the human DNA polymorphism discovery resource (Coriell Institute). For SEP53, methods were identical to those described for the orangutan and gorilla above. For p53, which has many small exons interspersed with intronic sequence, we placed 6 PCR primer pairs in introns such that we amplified and obtained sequences from exons 2 to 11. Following alignment and removal of intronic sequence, we used the 48 alleles obtained from each gene to perform MacDonald-Kreitman tests as implemented in DNAsp. These test for a departure from the neutral expectation that the ratio of non-synonymous to synonymous fixed differences between species will be the same as the ratio for polymorphism within species [39].

## Supporting Information

Table S1 (0.03 MB DOC)

Figure S1 Predicted amino acid sequence and alignment of SEP53 proteins from 9 mammalian species. The highly conserved N-terminal Ca+ binding domain spans amino acids 1 to 90. The diamond (⋄) designates the positions of the insertion sites of chimp, see figure 2. Porcine SEP53 also contain an insert in this region (Further details and functional analyses af porcine SEP53 are being presented elsewhere)
(5.24 MB TIF)

## References

[1] Mosser DD, Morimoto RI (2004). Molecular chaperones and the stress of oncogenesis.. Oncogene.

[2] Rutherford SL, Lindquist S (1998). Hsp90 as a capacitor for morphological evolution.. Nature.

[3] Blake MJ, Fargnoli J, Gershon D, Holbrook NJ (1991). Concomitant decline in heat-induced hyperthermia and HSP70 mRNA expression in aged rats.. Am J Physiol.

[4] Blake MJ, Gershon D, Fargnoli J, Holbrook NJ (1990). Discordant expression of heat shock protein mRNAs in tissues of heat-stressed rats.. J Biol Chem.

[5] Lakhotia SC, Singh BN (1996). Synthesis of a ubiquitously present new HSP60 family protein is enhanced by heat shock only in the Malpighian tubules of Drosophila.. Experientia.

[6] Kuper H, Adami HO, Trichopoulos D (2000). Infections as a major preventable cause of human cancer.. J Intern Med.

[7] Cox JT (2006). The development of cervical cancer and its precursors: what is the role of human papillomavirus infection?. Curr Opin Obstet Gynecol.

[8] Snijders PJF, Steenbergen RDM, Heideman DAM, Meijer C (2006). HPV-mediated cervical carcinogenesis: concepts and clinical implications.. Journal of Pathology.

[9] Hopwood D, Moitra S, Vojtesek B, Johnston DA, Dillon JF (1997). Biochemical analysis of the stress protein response in human oesophageal epithelium.. Gut.

[10] Yagui-Beltran A, Craig AL, Lawrie L, Thompson D, Pospisilova S (2001). The human oesophageal squamous epithelium exhibits a novel type of heat shock protein response.. Eur J Biochem.

[11] Imai FL, Uzawa K, Nimura Y, Moriya T, Imai MA (2005). Chromosome 1 open reading frame 10 (C1orf10) gene is frequently down-regulated and inhibits cell proliferation in oral squamous cell carcinoma.. Int J Biochem Cell Biol.

[12] Contzler R, Favre B, Huber M, Hohl D (2005). Cornulin, a new member of the “fused gene” family, is expressed during epidermal differentiation.. J Invest Dermatol.

[13] Darragh J, Hunter M, Pohler E, Nelson L, Dillon JF (2006). The calcium-binding domain of the stress protein SEP53 is required for survival in response to deoxycholic acid-mediated injury.. Febs Journal.

[14] Little TJ, Cobbe N (2005). The evolution of immune-related genes from disease carrying mosquitoes: diversity in a peptidoglycan- and a thioester-recognising protein.. Insect Molecular Biology.

[15] Little TJ, Colbourne JK, Crease TJ (2004). Molecular evolution of *Daphnia* immunity genes: polymorphism in a Gram Negative Binding Protein and an Alpha-2-Macroglobulin.. Journal of Molecular Evolution.

[16] Obbard D, Linton Y, Jiggins F, Yan G, Little T (2007). Population genetics of Plasmodium resistance genes in Anopheles gambiae: no evidence for strong selection.. Molecular Ecology.

[17] Obbard DJ, Jiggins FM, Little TJ (2006). Rapid Evolution of antiviral RNAi genes.. Current Biology.

[18] Ford MJ (2002). Applications of selective neutrality tests to molecular ecology.. Molecular Ecology.

[19] Hurst LD (2002). The Ka/Ks ratio:diagosing the form of sequence evolution.. Trends in Biochemical Sciences.

[20] Schlenke TA, Begun DJ (2003). Natural selection drives *Drosophila* immune system evolution.. Genetics.

[21] Wyckoff GJ, Malcom CM, Vallender EJ, Lahn BT (2005). A highly unexpected strong correlation between fixation probability of nonsynonymous mutations and mutation rate.. Trends in Genetics.

[22] Yang Z (1997). PAML: a program package for phylogenetic analysis by maximum likelihood.. Cabios.

[23] Yang Z, Bielawski JP (2000). Statistical methods for detecting molecular adaptation.. Trends in Ecology&Evolution.

[24] Yang Z, Nielsen R, Goldman N, Pedersen A-MK (2000). Codon-substitution models for heterogeneous selection pressure at amino acid sites.. Genetics.

[25] Clark NL, Swanson WJ (2005). Pervasive Adaptive Evolution in Primate Seminal Protiens.. Plos Biology.

[26] Karin M, Lawrence T, Nizet V (2006). Innate Immunity Gone Awry: Linking Microbial Infections to Chronic Inflammation and Cancer Cell.

[27] Brandtzaeg P, Baekkevold ES, Farstad IN, Jahnsen FL, Johansen FE (1999). Regional specialization in the mucosal immune system: what happens in the microcompartments?. Immunology Today.

[28] Eckmann L, Kagnoff MF (2005). Intestinal mucosal responses to microbial infection.. Springer Seminars in Immunopathology.

[29] Iimura M, Gallo RL, Hase K, Miyamoto Y, Eckmann L (2005). Cathelicidin mediates innate intestinal defense against colonization with epithelial adherent bacterial pathogens.. Journal of Immunology.

[30] Chae SW, Pothhoulakis C, Eckmann L, Kagnoff MF (2004). IKK beta dependent activation of NF-kappa B in intestinal epithelial cells has a protective role in Clostridium difficile toxin A-induced enteritis.. Gastroenterology.

[31] Li WH, Saunders MA (2005). The chimpanzee and us.. Nature.

[32] Patterson N, Richter DJ, Gnerre S, Lander ES, Reich D (2006). Genetic evidence for complex speciation of humans and chimpanzees.. Nature.

[33] Mikkelsen TS, Hillier LW, Eichler EE, Zody MC, Jaffe DB (2005). Initial sequence of the chimpanzee genome and comparison with the human genome.. Nature.

[34] Altschul SF, Madden TL, Schäffer AA, Zhang J, Zhang Z (1997). Gapped BLAST and PSI-BLAST: a new generation of protein database search programs.. Nucleic Acids Research.

[35] Rozas J, Rozas R (1999). DNAsp version 3: an integrated program for population genetics and molecular evolution analysis.. Bioinformatics.

[36] Yang Z, Wong WSW, Nielsen R (2005). Bayes empirical inference of amino acid sites under positive selection.. Molecular Biology and Evolution.

[37] Pond SK, Muse SV (2005). Site-to-site variation of synonymous substitution rates.. Molecular Biology and Evolution.

[38] Kosakovsky-Pond SL, Frost SDW, Muse SV (2005). HyPhy: hypothesis testing using phylogenies.. Bioinformatics.

[39] MacDonald J, Kreitman M (1991). Adaptive protein evolution at the *adh* locus in *Drosophila*.. Nature.

