# Supplementary material for: Adaptive Evolution of a Stress Response Protein
Source: PLoS One. 2007 Oct 10;2(10):e1003. doi: 10.1371/journal.pone.0001003 (PMC1994589; doi:10.1371/journal.pone.0001003)
Supplement: Table S1 — (0.03 MB DOC) [file pone.0001003.s001.doc]

Table S1 Numbers for Macdonald and Kreitman tests. ‘Polymorphic’ indicates polymorphic and is a count of synonymous and non-synonymous polymorphisms within species, while ‘Fixed’ is a count of fixed synonymous and non-synonymous nucleotide differences between species. p-values were calculated using Fisher’s exact test. Note that p-values are for a two-tailed test and the near significance for the P53 comparisons reflect a possible excess of non-synonymous polymorphism.

|  |  |  | Poly | |  |  | |  | | Fixed | | |  | |  |  |
| --- | --- | --- | --- | --- | --- | --- | --- | --- | --- | --- | --- | --- | --- | --- | --- | --- |
| Gene | Comparison | Syn |  | Non | | |  | | Syn | |  | Non | | p | | |
| SEP53 | Human vs Chimp | 3 |  | 3 | | |  | | 3 | |  | 8 | | 0.60 | | |
|  | Human vs Gorilla | 3 |  | 3 | | |  | | 3 | |  | 16 | | 0.12 | | |
|  | Human vs Orangutan | 3 |  | 3 | | |  | | 8 | |  | 30 | | 0.15 | | |
|  | Human vs Macaque | 3 |  | 3 | | |  | | 26 | |  | 50 | | 0.35 | | |
| P53 | Human vs Chimp | 0 |  | 3 | | |  | | 3 | |  | 0 | | 0.10 | | |
|  | Human vs macaque | 0 |  | 3 | | |  | | 24 | |  | 18 | | 0.09 | | |
