# Supplementary figures and images for: Adaptive Evolution of a Stress Response Protein
Source: PLoS One. 2007 Oct 10;2(10):e1003. doi: 10.1371/journal.pone.0001003 (PMC1994589; doi:10.1371/journal.pone.0001003)

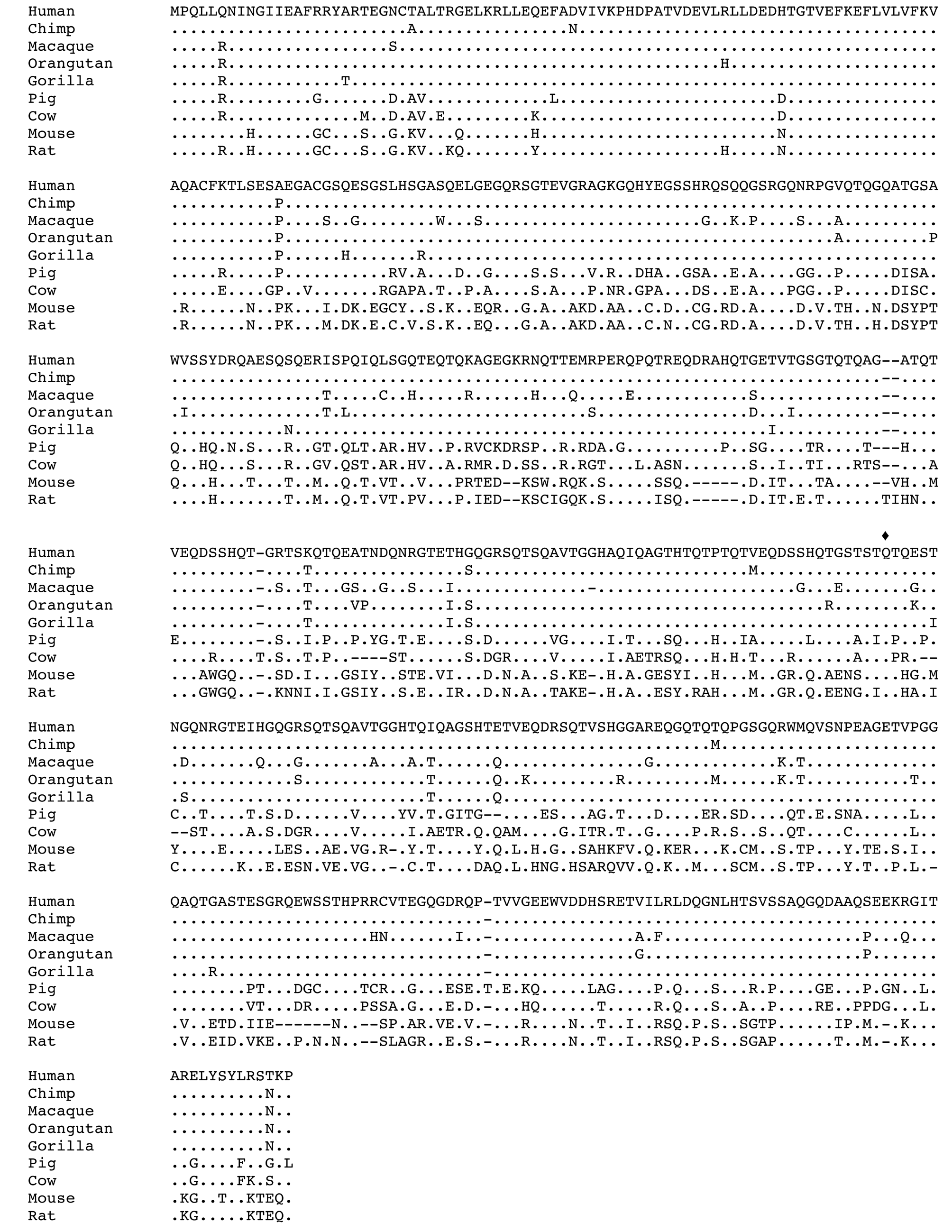

Supplement: Figure S1 — Predicted amino acid sequence and alignment of SEP53 proteins from 9 mammalian species. The highly conserved N-terminal Ca+ binding domain spans amino acids 1 to 90. The diamond (⋄) designates the positions of the insertion sites of chimp, see figure 2. Porcine SEP53 also contain an insert in this region (Further details and functional analyses af porcine SEP53 are being presented elsewhere) (5.24 MB TIF) [file pone.0001003.s002.tif]
